# First-Principles Study of the Stability, Electronic Structure, and Mechanical Properties of Ce-Doped MgZn_2_

**DOI:** 10.3390/ma19010050

**Published:** 2025-12-22

**Authors:** Jiaxing Guo, Hongyang Zhao, Zhanyi Hui, Lin Zhang, Hongyu Liu

**Affiliations:** 1 School of Mechanical Engineering and Automation, University of Science and Technology Liaoning, Anshan 114051, China; 18524331620@163.com (J.G.);; 2 School of Materials and Metallurgy, University of Science and Technology Liaoning, Anshan 114051, China

**Keywords:** Ce doping, electronic structure, mechanical properties, first-principles calculations

## Abstract

The structural stability, electronic structure, and elastic properties of MgZn_2_, Mg_3_Zn_8_Ce, and Mg_4_Zn_7_Ce have been investigated by adopting first-principles calculations methods based on density functional theory. The calculated lattice parameters agree well with experimental values and previous calculations. Formation enthalpy and binding energy calculations show that Mg_3_Zn_8_Ce has the highest alloying ability and structural stability. Electronic structure analysis suggests that Ce doping forms strong covalent bonds with Mg and Zn atoms, enhancing the stability of the system. Mechanical property calculations show that Mg_4_Zn_7_Ce exhibits the highest toughness, while Mg_3_Zn_8_Ce demonstrates the best shear resistance. Thus, Ce doping increases the stability and bonding strength of MgZn_2_, reduces material brittleness, and enhances material ductility. This computational analysis provides theoretical support for predicting the properties of Mg-Zn-Ce alloys.

## 1. Introduction

Magnesium alloys, recognized as lightweight structural materials with great potential, have seen a continuous rise in demand in high-end sectors such as aerospace and automotive industries due to their low density (approximately 1.74 g/cm^3^), high specific strength, and excellent recyclability. These properties make them essential for achieving energy conservation and emission reduction goals [1]. Among the various magnesium alloy systems, Mg–Zn alloys are considered one of the core development systems due to their favorable balance of strength and formability [2]. The most representative intermetallic compound in Mg–Zn alloys is MgZn_2_, which has garnered significant attention in materials science as a Laves phase. As a typical Laves phase, MgZn_2_ plays a critical role in strengthening the alloy. It adopts a hexagonal C14-type Laves structure with space group P6_3_/mmc, and its stability is closely linked to the Zn content [3]. In addition to MgZn_2_, other strengthening phases exist within the Mg–Zn alloy system, such as Mg_4_Zn_7_, Mg_2_Zn_11_, and Mg_21_Zn_25_, each exhibiting distinct crystal structures and varying stability [4]. Beyond Zn, rare earth elements, including Ce and La, are receiving increasing attention in Mg alloys due to their ability to improve microstructure and enhance mechanical properties. Specifically, Ce-doped MgZn_2_ has been shown to modulate its crystal structure and form new phases, which can significantly influence the alloy’s electronic structure, mechanical behavior, and high-temperature stability. The addition of Ce to Mg alloys improves both their strength and ductility, while also enhancing the stability and electronic characteristics of MgZn_2_ [4].

Experimentally, studies on the processing of Mg–Zn–Ce alloys have concluded that the addition of Ce promotes non-planar slip, weakens the planar texture, and enhances recrystallization, thereby influencing the precipitation phase behavior. Through this processing-microstructure coupling effect, Wu et al. demonstrated that Ce not only chemically alters the composition of the precipitation phases but also affects the nucleation and growth distribution of phases such as MgZn_2_ via plastic deformation and recrystallization pathways, thereby regulating their morphology and size [5]. The addition of Ce leads to the formation of Ce-containing compounds and alters lattice or interfacial energy conditions, which can suppress the precipitation, growth, and eutectoid transformation of the MgZn_2_ phase. This enhances the overall thermal stability of the precipitation structure and modifies the nucleation and growth pathways [6]. The addition of Ce to Mg–Zn alloys results in the formation of a Ce-rich secondary phase, which effectively “displaces” Zn from the matrix solution. This process reduces the available Zn for the formation of precipitates in the Mg–Zn system, such as MgZn_2_. As a result, Ce addition may inhibit the formation of MgZn_2_ precipitates by weakening the solute concentration gradient [7].

Although experimental studies have established a correlation between Ce doping and the evolution of the MgZn_2_ phase as well as alloy properties, several unresolved issues remain at the micro-mechanism level. First, the preferred occupancy of Ce atoms within the MgZn_2_ lattice (whether they replace Mg or Zn) and its impact on formation enthalpy are still unclear. Second, at the electronic structure level, direct evidence is lacking regarding the bonding type (ionic or covalent) between Ce and Mg/Zn atoms and the charge transfer mechanism—key factors that could explain the differences in phase stability. Third, the mechanical properties of the alloy, as experimentally measured, result from multiple factors, including grain refinement and second-phase strengthening. Isolating intrinsic parameters, such as the elastic modulus and hardness of the MgZn_2_ phase itself, is difficult, making it challenging to quantify the direct regulatory effect of Ce doping on the mechanical properties of the strengthening phase.

First-principles calculations provide an effective approach to address the aforementioned experimental bottlenecks. This method enables the construction of atomic-level models to precisely calculate thermodynamic parameters such as formation enthalpy and binding energy in doped systems, thereby clarifying the stability criteria for Ce-doped MgZn_2_ phases. Chen Jingchang et al. [8] investigated the influence mechanism of Y doping on the MgZn_2_ phase. Based on computational results, they concluded that Y doping enhances the stability, bond strength, and fracture toughness of MgZn_2_. Jin et al. [9] employed first-principles calculations to study the effects of Y and Ca microalloying on the Guinier-Preston region and Guinier-Preston 1 regions of Mg-Zn alloys: Y enhances the strength and hardness of the single atomic layer Guinier-Preston region but reduces ductility; Ca alters its Young’s modulus anisotropy; Y-Ca compounding significantly modifies the shear modulus anisotropy of this phase. Concurrently, density of states and charge density difference analysis can visually reveal the electronic hybridization characteristics between Ce atoms and neighboring atoms, elucidating the essence of bonding strength changes. Elastic constant calculations directly yield intrinsic mechanical parameters such as Young’s modulus and shear modulus of the MgZn_2_ phase, establishing quantitative relationships between doping concentration and the mechanical properties of strengthening phases.

In summary, this study investigates the formation enthalpy, binding energy, density of states, and elastic constants of the MgZn_2_ phase—the key strengthening phase in Mg-Zn-Ce alloys—under different Ce atomic occupancy patterns using first-principles calculations. This research provides theoretical support for predicting the properties of Mg-Zn-Ce alloys.

## 2. Theoretical Models and Computational Methods

**Figure 1:**
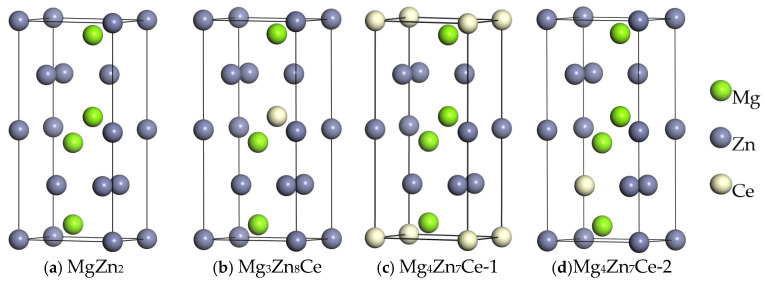
Schematic diagrams of the crystal structures of MgZn_2_, Mg_3_Zn_8_Ce, Mg_4_Zn_7_Ce-1, and Mg_4_Zn_7_Ce-2.

The MgZn_2_ phase belongs to the hexagonal crystal system with space group P63/MMC [10]. The unit cell contains 12 atoms, including 4 Mg atoms and 8 Zn atoms, with lattice constants a = b = 5.25 Å, c = 8.73 Å, α = β = 90°, and γ = 120°, as shown in Figure 1a. Since the atomic radius of Ce is significantly larger than those of Mg and Zn, as documented in [11], this corresponds to substitution doping. Within the MgZn_2_ unit cell, Mg atoms occupy only one equivalent site, while Zn atoms occupy two equivalent sites. Thus, replacing one Mg atom or one Zn atom with one Ce atom yields three substitution solid solutions: Mg_3_Zn_8_Ce, Mg_4_Zn_7_Ce-1, and Mg_4_Zn_7_Ce-2 (where 1 and 2 denote the first and second equivalent sites of Zn, respectively), as depicted in Figure 1b–d. Figure 1 shows the crystal structure of MgZn_2_.

Calculations were performed using the Castep (Cambridge Serial Total Energy Package) module within Materials Studio 2023 software [12], The Castep module is a density functional theory-based ab initio quantum mechanical software. The exchange correlation potential was considered by the generalized gradient approximation (GGA) in the scheme of Perdew–Burke–Eruzerhof (PBE) [13]. Ultrasoft pseudopotentials [14] were employed to describe valence electron-ion interactions, with a kinetic energy cutoff of 481 eV and a K-point grid of 6 × 6 × 3. Geometric optimization was carried out using the BFGS method [15]. In the relaxation procedure, every atom is allowed to reach its most stable local configuration, with all parameters selected with the highest level of precision. During the self-consistent field (SCF) calculations, the stress deviation was maintained below 0.02 GPa. The total energy convergence criterion was set to 5 × 10^−^^7^ eV/atom, the force convergence criterion to 0.01 eV/Å, and the displacement convergence criterion to 0.0005 Å. The Pulay mixing scheme was applied.

## 3. Results and Discussion

### 3.1. Crystal Structure and Stability

The equilibrium lattice constants and unit cell volumes of MgZn_2_ after doping optimization are shown in Table 1. The deviation between the calculated MgZn_2_ lattice constants and those reported in [16] is less than 1%, demonstrating the accuracy of this calculation. Doping Ce into the MgZn_2_ unit cell alters the lattice type, lattice constant, and unit cell volume due to the larger atomic radius of the rare-earth element Ce compared to Mg and Zn. This demonstrates that doping with the rare-earth element Ce can modify the structural parameters of MgZn_2_.

Based on structural optimization, the energy relationships of the three alloys were first analyzed, primarily encompassing alloy formation enthalpy and binding energy. Formation enthalpy represents the energy absorbed or released during alloy formation, indicating the strength of the alloy’s compound formation capability. When *H_form_* < 0, a lower formation enthalpy facilitates easier alloy compound formation, while a higher formation enthalpy makes alloy compound formation more difficult. Binding energy reflects the stability of the crystal structure. A higher absolute value of binding energy indicates greater stability in the crystal structure and stronger binding forces between atoms within the crystal [18]. The calculation formulas for alloy formation enthalpy and binding energy [19] are as follows:

(1) $$H_{form}=\frac{E_{tot}-N_{A}E_{\mathrm{solid}}^{A}-N_{B}E_{solid}^{B}-N_{C}E_{solid}^{C}}{N_{A}+N_{B}+N_{C}}$$

(2) $$E_{coh}=\frac{E_{tot}-N_{A}E_{atom}^{A}-N_{B}E_{atom}^{B}-N_{C}E_{atom}^{C}}{N_{A}+N_{B}+N_{C}}$$

In the equation: $N_{A}$, $N_{B}$, $N_{C}$ represent the number of atoms of elements A, B, and C in the compound’s unit cell, respectively; $E_{tot}$ is the total energy of the compound; $E_{solid}^{A}$, $E_{solid}^{B}$, $E_{solid}^{C}$ represent the average energy per atom of solid elements A, B, and C, respectively. The calculated values for the single-atom energies of Mg, Zn, and Ce crystals are: −973.960 eV, −1709.755 eV, and −1061.976 eV; $E_{atom}^{A}$, $E_{atom}^{B}$, $E_{atom}^{C}$ denote the energies of A, B, and C atoms in their isolated states. The calculated values for the free atom energies of Mg, Zn, and Ce are −972.330 eV, −1708.621 eV, and −1057.154 eV, respectively. The formation enthalpies and binding energies for MgZn_2_, Mg_3_Zn_8_Ce, Mg_4_Zn_7_Ce-1, and Mg_4_Zn_7_Ce-2 are listed in Table 2.

As shown in Table 2, the formation enthalpies of the four compounds are all negative, indicating that all four alloy compounds can form spontaneously. Mg_3_Zn_8_Ce exhibits the lowest formation enthalpy, making it the most easily formed alloy compound with strong alloying ability, suggesting that Ce atoms are more likely to replace Mg atoms. In contrast, Mg_4_Zn_7_Ce-2 exhibits the highest formation enthalpy, indicating that it is the least likely to form alloy compounds, with weaker alloying ability. As shown in Table 2, the formation enthalpies of MgZn_2_ and the three solid solution alloys are all negative, meaning the formation processes of these compounds are exothermic, allowing them to exist stably. Moreover, Mg_3_Zn_8_Ce exhibits the highest absolute value of formation enthalpy, indicating better thermal stability and a higher melting point. Mg_4_Zn_7_Ce-2 exhibits the lowest absolute value of formation enthalpy, indicating poorer thermal stability and a lower melting point. The binding energy increases from MgZn_2_, Mg_4_Zn_7_Ce-2, Mg_4_Zn_7_Ce-1, to Mg_3_Zn_8_Ce, implying that MgZn_2_ exhibits the lowest structural stability, while Mg_3_Zn_8_Ce demonstrates the highest structural stability. Given the lower alloying capacity and structural stability of Mg_4_Zn_7_Ce-2, the probability of intermediate phase formation when doped with Ce is low, and therefore, it was excluded from subsequent calculations.

### 3.2. Electronic Structure

**Figure 2:**
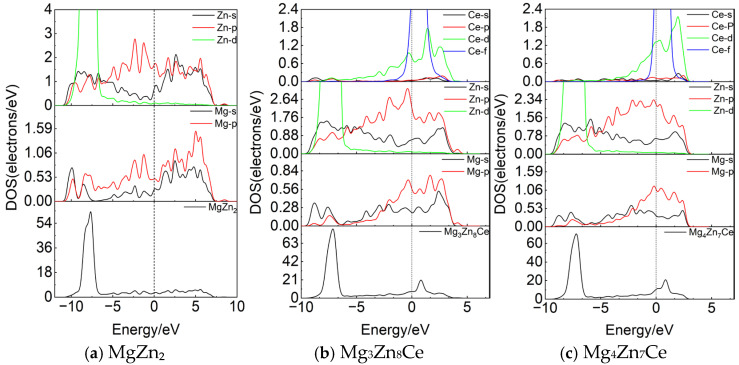
Electronic density of states of MgZn_2_, Mg_3_Zn_8_Ce, and Mg_4_Zn_7_Ce.

The density of states structure reflects the electronic structure of materials, providing insights into electron bonding and interactions within the system. It serves as a crucial parameter determining the structural stability and electrical conductivity of materials [22]. The electronic density of states curves for MgZn_2_, Mg_3_Zn_8_Ce, and Mg_4_Zn_7_Ce are shown in Figure 2. The dashed line indicates the Fermi level, corresponding to the zero-energy point. The valence electron configurations for each element are as follows: Mg 2p^6^3s^2^, Zn 3p^6^3d^10^4s^2^ and Ce 4f^1^5d^16^s^2^.

As shown in Figure 2, the bonding electrons in MgZn_2_, Mg_3_Zn_8_Ce, and Mg_4_Zn_7_Ce are primarily distributed within the energy ranges of −11 to 6 eV, −10 to 5 eV, and −10 to 3 eV, respectively. MgZn_2_ shows no band gap around the Fermi level, indicating metallic behavior consistent with the typical characteristics of the Laves phase [23]. The total density of states (TDOS) in the valence band is divided into two main regions. In the energy range from −10 eV to −5 eV, the primary bonding peak originates from the Zn 3d orbital, which contributes most significantly to the TDOS in this region. In the region from −5.0 eV to the Fermi level, the TDOS is predominantly attributed to the Mg 3s and 2p orbitals, along with the Zn 3p orbital. Above the Fermi level, weak hybridization occurs between the Zn 4s orbital and the Mg sp orbitals, suggesting the presence of covalent bonding in the MgZn_2_ crystal structure. In the bonding region of Mg_3_Zn_8_Ce, partial hybridization between the Ce 5d orbital and the Mg 2p orbital indicates covalent bonding between Ce and Mg atoms. Additionally, the Ce 5d and Zn 3p orbitals exhibit significant hybridization, suggesting strong covalent bonding between Ce and Zn atoms. In the bonding region of Mg_4_Zn_7_Ce, hybridization between the Ce 5d and Mg 2p orbitals indicates covalent bonding between Ce and Mg atoms, while hybridization between Ce and Zn orbitals is negligible, indicating that Ce and Zn atoms do not form significant covalent bonds.

**Figure 3:**
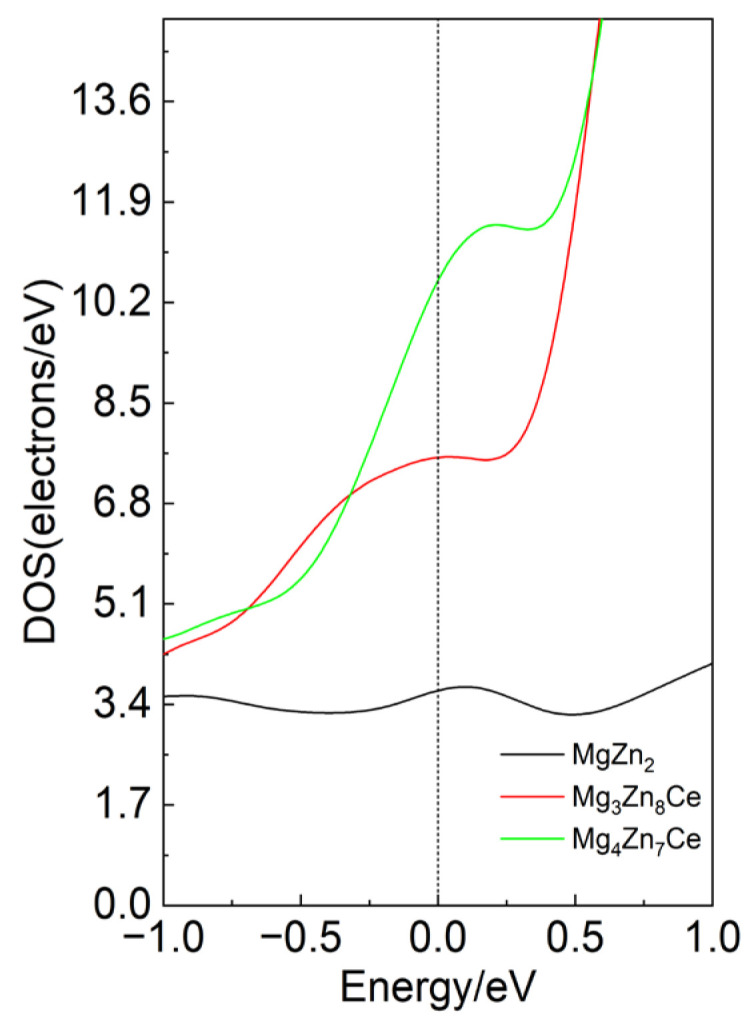
Total density states of MgZn_2_, Mg_3_Zn_8_Ce and Mg_4_Zn_7_Ce.

The total density of states at the Fermi level reflects the metallic character of the doped system [24]. As shown in Figure 3 for MgZn_2_, Mg_3_Zn_8_Ce, and Mg_4_Zn_7_Ce, the total density of states at the Fermi level decreases in the order Mg_4_Zn_7_Ce, Mg_3_Zn_8_Ce, and MgZn_2_. This indicates that Ce doping contributes to enhancing the metallic character of MgZn_2_. The enhanced density of states (DOS) near the Fermi level may improve the electrical conductivity of MgZn_2_ alloys. An increased DOS indicates a higher availability of electronic states for conduction electrons, which can enhance electron mobility and, consequently, the material’s electrical conductivity. This property suggests that MgZn_2_ alloys may be more suitable for applications in electronic devices and conductive materials, particularly in microelectronics and battery electrode materials, where high conductivity is crucial.

### 3.3. Mulliken Charge Analysis

Through density functional analysis, the distribution and charge transfer in atomic orbitals can be determined, thereby establishing the bonding relationships between atoms [25]. The absolute value of the bond overlap density provides insight into the bond strength between atoms, with a higher overlap density indicating a stronger bond. A positive bond overlap density is generally associated with covalent bonding, while a negative or highly asymmetric bond overlap density suggests an ionic character [26]. The electron occupancy numbers of MgZn_2_, Mg_3_Zn_8_Ce, and Mg_4_Zn_7_Ce are shown in Table 3. In MgZn_2_, the bond order between Mg and Zn atoms is 0.07, suggesting the formation of weak covalent bonds. In contrast, the bond order between Zn atoms is 0.28, indicating the presence of stronger covalent bonds between Zn atoms. In Mg_3_Zn_8_Ce, the charge on the Ce ion is +1.022, reflecting a significant positive charge and demonstrating the strong electronegativity of Ce within the alloy. The electron distribution of Ce is relatively broad, involving both d and f orbitals, which facilitates its significant role in bonding, particularly in interactions with Mg and Zn atoms. The bond order between Ce and Zn atoms is 0.51, signifying the formation of a strong covalent bond between Ce and Zn. The bond order between Ce and Mg atoms approaches zero, indicating a relatively weak interaction between these atoms. In Mg_4_Zn_7_Ce, the bond order between Zn atoms is 0.97, signifying the formation of strong covalent bonds between Zn atoms. The bonding between Ce and Mg atoms is strong, primarily due to the high electronegativity of the Ce ion, which has a Hirshfeld charge of −0.13. In contrast, the bonding between Ce and Zn is weak, with a low bond order, suggesting that the interaction between Ce and Zn is predominantly ionic. The bond order between Mg and Zn atoms is −0.04, reflecting a relatively weak interaction. The bond length of 2.92 Å is relatively long, and the electron density is low, further supporting the conclusion that the Mg-Zn bond is ionic in nature.

## 4. Mechanical Properties

The elastic constants *C_ij_* are commonly used to characterize an alloy’s resistance to deformation under external forces, and play a crucial role in determining mechanical properties. The hexagonal crystal system comprises five independent elastic constants [27]: *C*_11_, *C*_12_, *C*_13_, *C*_33_, and *C*_44_. The calculated independent elastic constants for Ce-doped compounds are presented in Table 4. For hexagonal crystals, the criteria for mechanical stability are [28]: *C*_44_ > 0, *C*_11_ > |*C*_12_|, and (*C*_11_ + 2*C*_12_)*C*_33_ > $2C_{13}^{2}$. As shown in Table 4, all three compounds—MgZn_2_, Mg_3_Zn_8_Ce, and Mg_4_Zn_7_Ce—satisfy these mechanical stability criteria. When *C*_12_ − *C*_44_ > 0, the material exhibits ductility; when *C*_12_ − *C*_44_ < 0, it exhibits brittleness [29]. Based on the data in Table 4, the *C*_12_ − *C*_44_ values for these three compounds are greater than zero, indicating that all three compounds exhibit ductility.

The bulk modulus *B* and shear modulus *G* of the above three compounds were calculated using the VRH [32] approximation method, as shown in Equations (3)–(10).

(3) $$B_{\nu }=(\frac{1}{9})[C_{11}+C_{22}+C_{33}+2(C_{12}+C_{13}+C_{23})]$$

(4) $$G_{v}=(1/30)(M+12C_{44}+12C_{66})$$

(5) $$B_{R}=\frac{C^{2}}{M}$$

(6) $$G_{R}=\frac{\left(\frac{5}{2})[C^{2}C_{44}C_{66}\right]}{\left[3B_{V}C_{44}C_{66}+C^{2}(C_{44}+C_{66})\right]}$$

(7) $$M=C_{11}+C_{12}+2C_{33}-4C_{13}$$

(8) $$C^{2}=(C_{11}+C_{12})C_{33}-2C_{13}^{2}$$

(9) $$B_{H}=\frac{1}{2}(B_{R}+B_{V})$$

(10) $$G_{H}=\frac{1}{2}(G_{R}+G_{V})$$

Based on *B* and *G*, the elastic modulus *E*, Poisson’s ratio *υ*, and Vickers hardness *H_υ_* of polycrystalline materials can be calculated, as shown in Equations (11)–(13).

(11) $$E=\frac{9BG}{3B+G}$$

(12) $$\nu =\frac{3B-2G}{2(3B+G)}$$

(13) $$H_{v}=\frac{(1-2\nu )E}{6(1+\nu )}$$

The bulk modulus *B* reflects a material’s resistance to deformation under applied stress; with a higher value indicates stronger deformation resistance [33]. As shown in Table 5, Mg_3_Zn_8_Ce exhibits the highest bulk modulus and thus the best deformation resistance, followed by MgZn_2_, while Mg_4_Zn_7_Ce shows the poorest deformation resistance. Ce doping provides only a marginal improvement in the deformation resistance of MgZn_2_. The ratio of shear modulus *G* to bulk modulus *B* quantifies a material’s ductility versus brittleness. When (*G/B*) exceeds 0.57, the phase is brittle; when (*G/B*) falls below 0.57, the phase is ductile. Calculating the *C*_12_ − *C*_44_ value also characterizes ductility versus brittleness: positive values signify ductile behavior, whereas negative values indicate brittleness. All three compounds exhibit positive *C*_12_ − *C*_44_ values, consistent with the conclusions drawn from the (G/B) criterion. Table 5 shows that the (*G/B*) values of all three compounds are below 0.57. That is, all three compounds exhibit ductility. Mg_4_Zn_7_Ce exhibits the highest ductility, corresponding to the lowest elastic modulus *E*, which characterizes material stiffness. The shear modulus *G* and Poisson’s ratio υ characterize a material’s resistance to shear deformation. Higher *G* and lower υ indicate stronger shear resistance. Mg_3_Zn_8_Ce exhibits the highest *G* and lowest *υ*, demonstrating superior shear resistance.

## 5. Discussion on the Mechanism and Engineering Significance of Toughness Enhancement

The primary motivation for investigating the Ce-doped MgZn_2_ phase lies in addressing the intrinsic brittleness of the Laves phase, which significantly limits the application of Mg-Zn series alloys. Similarly to the issues observed in 7xxx series aluminum alloys (Al-Zn-Mg-Cu), where the continuous enrichment of the hard MgZn_2_ phase at grain boundaries leads to stress concentration and intergranular fracture [35,36], the segregation of coarse MgZn_2_ in magnesium alloys often deteriorates fracture toughness. Experimental studies have shown that rare-earth (RE) microalloying is an effective strategy for improving microstructure and enhancing mechanical properties. The addition of trace rare-earth elements (such as Y or Ce) has been shown to reduce crack initiation sites and significantly increase the elongation of zinc-containing alloys [37,38]. Current research involves extremely low doping concentrations, and traditional experimental methods using a “trial-and-error” approach are insufficient to accurately characterize the specific influence mechanism of trace elements on the MgZn_2_ phase at the atomic scale. The results of first-principles calculations show that Ce substitution significantly reduces the shear-to-bulk modulus ratio (*G*/*B*) of the pure MgZn_2_ phase, indicating a transition from brittle to ductile behavior. Furthermore, the thermodynamic stability analysis confirms that the formation of the Ce-doped structure (Mg_3_Zn_8_Ce) is energetically favorable compared to the pure binary phase. The Ce doping model in this study can represent the energy-preferred local atomic environment inside the precipitated phase during the microalloying process. The mechanism revealed here suggests that Ce does not merely exist as a separate phase but stabilizes the MgZn_2_ lattice while simultaneously softening its elastic response.

## 6. Conclusions

(1) Calculations of formation enthalpy and binding energy indicate that Ce doping significantly enhances the structural stability of MgZn_2_. Notably, Mg_3_Zn_8_Ce exhibits the lowest formation enthalpy, suggesting a strong thermodynamic driving force for the formation of Ce-modified precipitates, which is favorable for stabilizing the strengthening phase in practical alloys.

(2) Electronic structure analysis reveals that Ce doping in MgZn_2_ induces the formation of strong covalent bonds between Ce-Zn and Ce-Mg atoms, thereby enhancing the stability of the MgZn_2_ system.

(3) Mechanical property calculations show that Ce doping effectively reduces the intrinsic brittleness of the MgZn_2_ phase, promoting the transition from brittle to ductile. This transition has significant engineering implications because it helps alleviate stress concentration caused by hard precipitates. Mg_4_Zn_7_Ce exhibits the highest toughness, while Mg_3_Zn_8_Ce shows the best shear resistance. By elucidating these atomic-scale mechanisms, this computational analysis provides theoretical support for predicting the performance of Mg-Zn-Ce alloys.

## Figures and Tables

**Table 1:** Equilibrium lattice constants and cell volumes after MgZn_2_ and Ce doping optimization.

Phase	Reference	Crystal System	*a*_0_ (Å)	*c*_0_ (Å)	*c*_0_/*a*_0_	*V*_0_ (Å^3^)
MgZn_2_	Present		5.222	8.595	1.645	202.979
	Exp. [16]	Hexagonal	5.222	8.568	1.640	202.341
	Cal. [17]		5.198	8.554	1.645	200.171
Mg_3_Zn_8_Ce	Present	Trigonal	5.257	9.008	1.714	215.593
Mg_4_Zn_7_Ce-1	Present	Trigonal	5.195	10.136	1.951	236.901
Mg_4_Zn_7_Ce-2	Present	Orthorhombic	5.633	8.763	1.556	240.863

**Table 2:** Formation enthalpy and binding energy calculation results.

Phase		*H_form_*/(kJ·mol^−1^)	*E_coh_*/(kJ·mol^−1^)
MgZn_2_	Present	−13.025	−138.375
	Cal. [20]	−13.346	−132.628
	Cal. [21]	−10.90	−139.60
Mg_3_Zn_8_Ce	Present	−17.234	−168.250
Mg_4_Zn_7_Ce-1	Present	−8.310	−158.840
Mg_4_Zn_7_Ce-2	Present	−1.068	−153.935

**Table 3:** Electron occupancy numbers of MgZn_2_, Mg_3_Zn_8_Ce, and Mg_4_Zn_7_Ce.

	Orbits						
Phase	Atom	s	p	d	f	Total	Charge(e)
MgZn_2_	Mg	0.515	6.471	0.000	0.000	6.896	1.014
	Zn(Ⅰ)	0.798	1.778	9.950	0.000	12.525	−0.525
	Zn(Ⅱ)	0.729	1.774	9.950	0.000	12.453	−0.453
Mg_3_Zn_8_Ce	Mg	0.539	6.453	0.000	0.000	6.992	1.008
	Zn(Ⅰ)	0.802	1.698	9.948	0.000	12.448	−0.448
	Zn(Ⅱ)	0.816	1.719	9.949	0.000	12.484	−0.484
	Ce	2.261	5.863	2.190	1.071	11.385	0.615
Mg_4_Zn_7_Ce	Mg	0.519	6.446	0.000	0.000	6.965	1.035
	Zn(Ⅰ)	0.776	1.731	9.951	0.000	12.458	−0.458
	Zn(Ⅱ)	0.660	1.625	9.954	0.000	12.239	−0.239
	Ce	2.465	6.260	2.055	1.118	11.897	0.103

**Table 4:** Independent elastic constants of MgZn_2_, Mg_3_Zn_8_Ce, and Mg_4_Zn_7_Ce.

Phase		*C*_11_	*C*_12_	*C*_13_	*C*_33_	*C*_44_
MgZn_2_	Present	90.280	62.976	27.683	124.624	25.390
	Cal. [30]	92	62	37	126	24
	Exp. [31]	107.25	45.45	27.43	126.40	27.70
Mg_3_Zn_8_Ce	Present	97.676	48.833	43.610	86.175	28.954
Mg_4_Zn_7_Ce	Present	84.304	40.462	34.133	58.856	9.275

**Table 5:** Volume modulus *B*, shear modulus *G*, Young’s modulus *E*, *G*/*B*, Poisson’s ratio *υ*, and Vickers hardness *H_υ_* of MgZn_2_, Mg_3_Zn_8_Ce, and Mg_4_Zn_7_Ce.

Phase.		*B*/GPa	*G*/GPa	*E*/GPa	*G*/*B*	*υ*	$H_{v}/\mathrm{GPa}$
MgZn_2_	Present	60.207	19.627	53.110	0.326	0.353	1.923
	Cal. [30]	60.61	22.52	60.53	0.35	0.34	
	Exp. [34]	70.71	16.12	45.57	0.23	0.39	
Mg_3_Zn_8_Ce	Present	61.300	25.918	68.149	0.423	0.315	3.195
Mg_4_Zn_7_Ce	Present	48.377	14.830	40.365	0.306	0.361	1.374

## Data Availability

The original contributions presented in this study are included in the article. Further inquiries can be directed to the corresponding author.
